# Supplementary material for: Extensive Therapeutic Drug Monitoring of Colistin in Critically Ill Patients Reveals Undetected Risks
Source: Microorganisms. 2020 Mar 15;8(3):415. doi: 10.3390/microorganisms8030415 (PMC7143967; doi:10.3390/microorganisms8030415)
Supplement: Supplementary file 1 [file microorganisms-08-00415-s001.pdf]

Extensive Therapeutic Drug Monitoring Of Colistin In Critically Ill Patients Reveals Undetected Risks.

Supplemental material 1 – elimination half times of Colistin

Patient 1

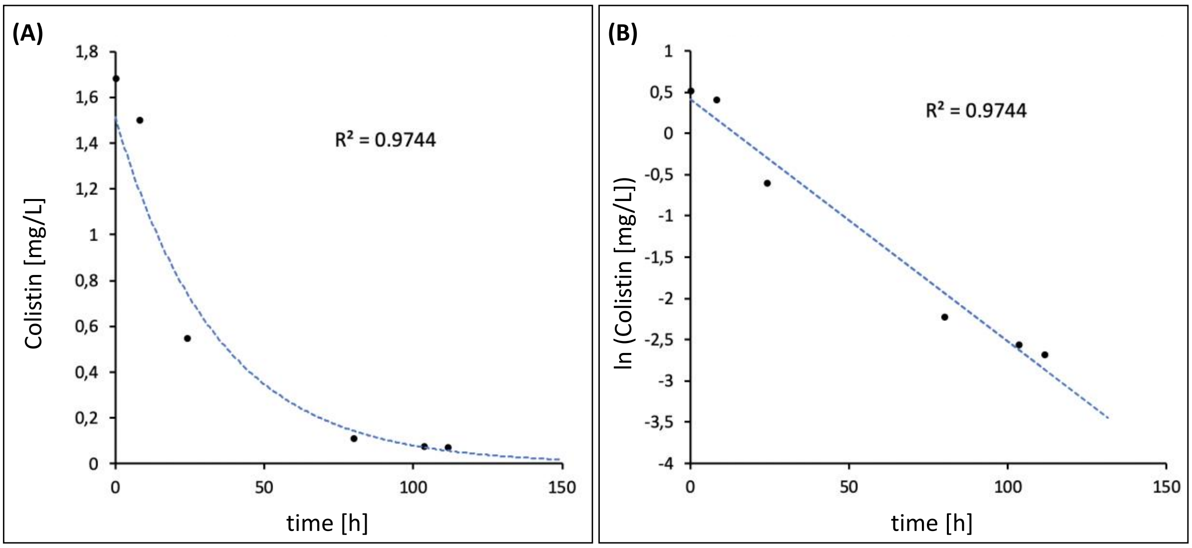

Figure S1: plasma levels of Colistin after cessation of CMS administration. (A) linear scaling of colistin plasma levels in [mg/L]; fit of an 1st decay elimination curve, $y = 1{,}5111e^{-0{,}029x}$, R^2= 0.97, p<0.001; (B) linear scaling colistin plasma levels in [mg/L] after logarithmic transformation; linear fit, y = -0,0294x + 0,4129, R^2= 0.97, p<0.001

Elimination half time: t1/2 = ln(0.5)/0.0294 = 23.6 [h]

Patient 2

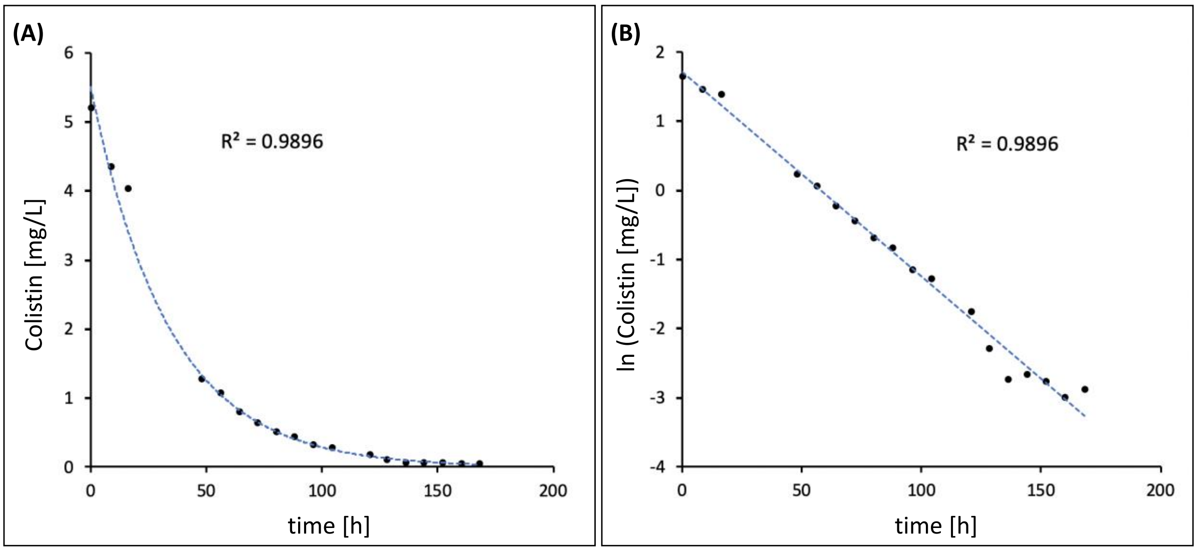

Figure S2: plasma levels of Colistin after cessation of CMS administration. (A) linear scaling of colistin plasma levels in [mg/L]; fit of an 1st decay elimination curve, y = $5{,}5021e^{-0{,}03x}$, R^2= 0.99, p<0.001; (B) linear scaling colistin plasma levels in [mg/L] after logarithmic transformation; linear fit, y = -0,0296x + 1,7051, R^2= 0.99, p<0.001

Elimination half time: t1/2 = ln(0.5)/0.0296 = 23.43 [h]

Patient 4

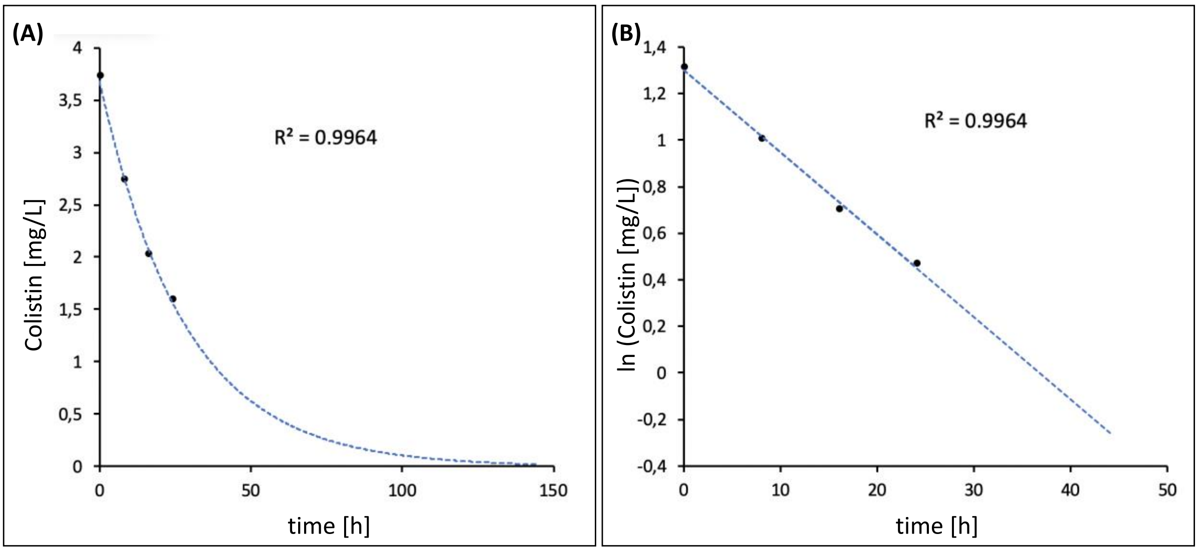

Figure S3: plasma levels of Colistin after cessation of CMS administration. (A) linear scaling of colistin plasma levels in [mg/L]; fit of an 1st decay elimination curve, $y = 3{,}73e^{-0{,}036x}$, R^2= 0.996, p<0.001; (B) linear scaling colistin plasma levels in [mg/L] after logarithmic transformation; linear fit, y = -0,0355x + 1,3022, R^2= 0.996, p<0.001

Elimination half time: t1/2 = ln(0.5)/0.0355 = 19.5 [h]

Patient 5

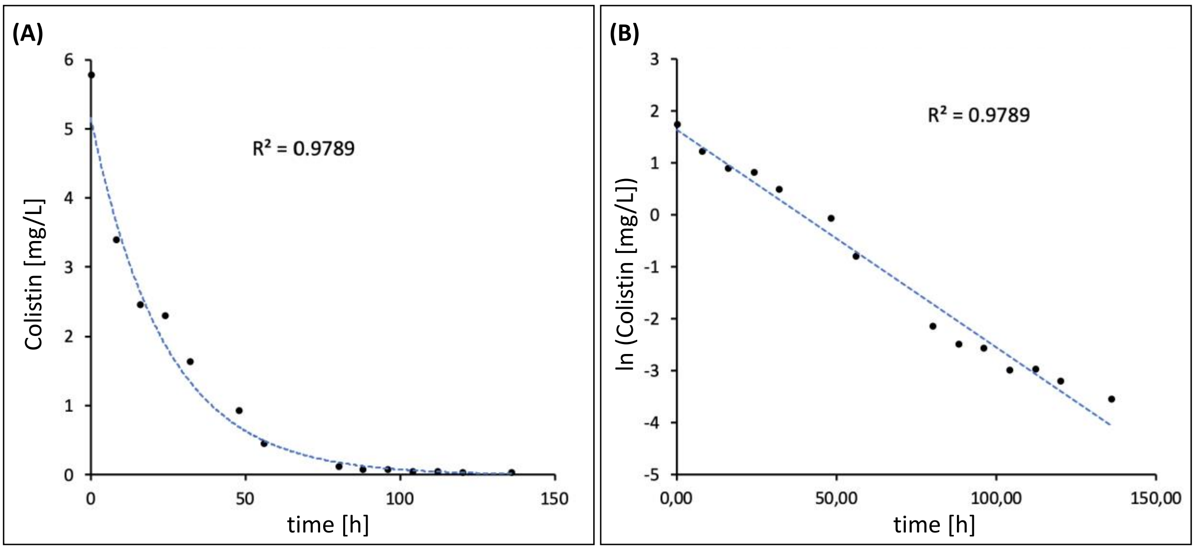

Figure S4: plasma levels of Colistin after cessation of CMS administration. (A) linear scaling of colistin plasma levels in [mg/L]; fit of an 1st decay elimination curve, $y = 5{,}1619e^{-0{,}042x}$, R^2= 0.98, p<0.001; (B) linear scaling colistin plasma levels in [mg/L] after logarithmic transformation; linear fit, y = -0,0419x + 1,6413, R^2= 0.98, p<0.001

Elimination half time: t1/2 = ln(0.5)/0.0419 = 16.5 [h]

Patient 6

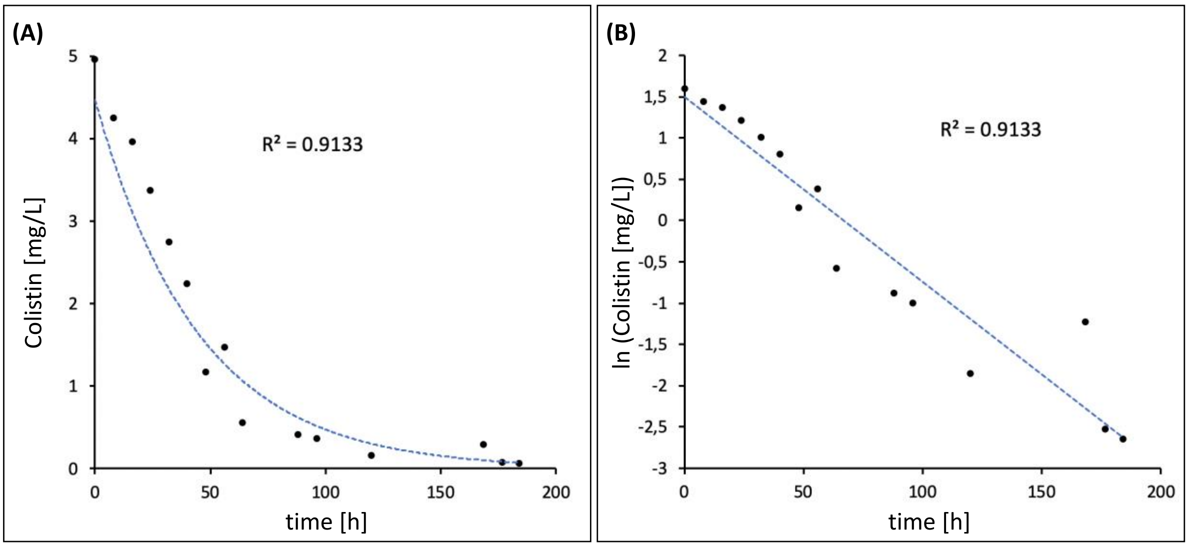

Figure S5: plasma levels of Colistin after cessation of CMS administration. (A) linear scaling of colistin plasma levels in [mg/L]; fit of an 1st decay elimination curve, $y = 4{,}96e^{-0{,}023x}$, R^2= 0.91, p<0.001; (B) linear scaling colistin plasma levels in [mg/L] after logarithmic transformation; linear fit, y = -0,0224x + 1,4961, R^2= 0.91, p<0.001

Elimination half time: t1/2 = ln(0.5)/0.0224 = 30.9 [h]

Patient 8

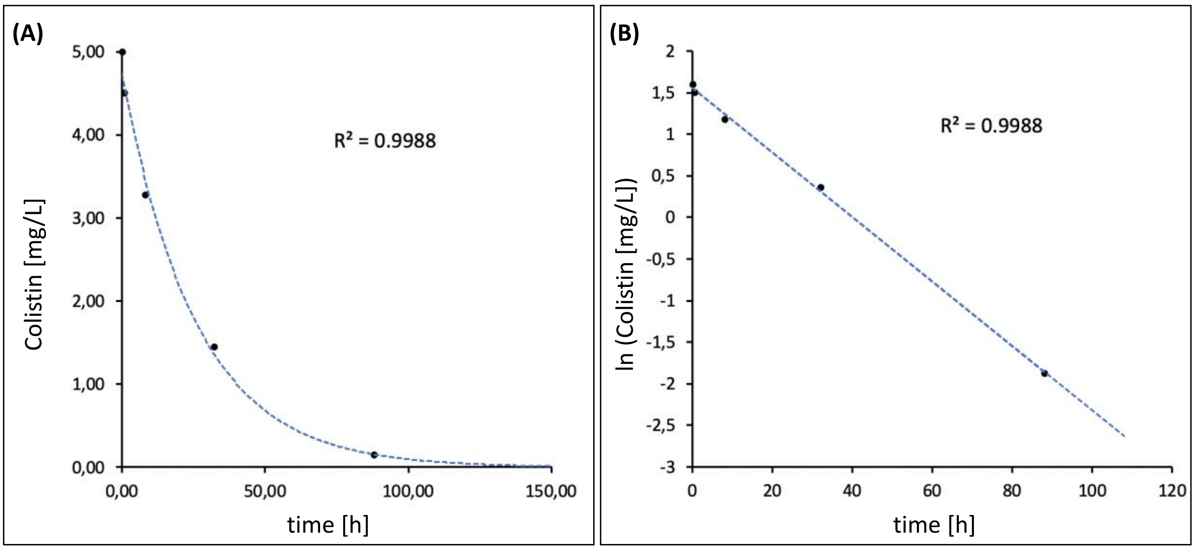

Figure S6: plasma levels of Colistin after cessation of CMS administration. (A) linear scaling of colistin plasma levels in [mg/L]; fit of an 1st decay elimination curve, y = $4,735e^{-0,039x}$, R^2= 0.999, p<0.001; (B) linear scaling colistin plasma levels in [mg/L] after logarithmic transformation; linear fit, y = -0,0387x + 1,555, R^2= 0.999, p<0.001

Elimination half time: t1/2 = ln(0.5)/0.0387 = 17.9 [h]
